# What Is Justice? Perspectives of Victims-Survivors of Gender-Based Violence

**DOI:** 10.1177/10778012231214772

**Published:** 2023-11-19

**Authors:** Marianne Hester, Emma Williamson, Nathan Eisenstadt, Hilary Abrahams, Nadia Aghtaie, Lis Bates, Geetanjali Gangoli, Amanda Robinson, Sarah-Jane Walker, Elizabeth McCarthy, Andrea Matolcsi, Natasha Mulvihill

**Affiliations:** 1 Centre for Gender and Violence Research, School for Policy Studies, 1980 University of Bristol, Bristol, UK; 2 Crime and Intelligence Innovation Institute, 2112 Cardiff University, Cardiff, UK; 3 101845 Women's Aid Federation England, Bristol, UK

**Keywords:** gender-based violence, justice systems, victim-survivor perspectives, inequalities

## Abstract

This article explores “how do victims-survivors of gender-based violence (GBV) experience and perceive justice?” based on interviews with 251 victims-survivors with experience of different types of GBV and criminal, civil, and family justice systems. Victims-survivors were found to have multiple perceptions of justice, related to different points in their journey following abuse and regarding individual, community, and societal responses. Perceptions relate to accountability; fairness in outcome and process; protection from future harm; recognition; agency; empowerment; affective justice; reparation; and social transformation. Current understandings of justice in legislative and policy approaches reproduce the “justice gap” by failing to take account of how survivors themselves understand and demand justice.

## Introduction

The research on which this article is based is rooted in the question: “how do victims-survivors of gender-based violence (GBV) experience and perceive justice”? For many years feminist scholars and activists have been concerned with whether engagement with criminal, civil, and/or family justice systems can provide safety for victims-survivors who have experienced domestic, sexual or “honor”-based violence. In countries such as England and Wales engagement with justice systems have tended to result in impunity with extremely low conviction rates for both domestic and sexual violence (Hohl & Stanko, 2015; ONS, 2020), the use of civil justice protection orders has been decreasing (Bates & Hester, 2020) and victim-survivors continue to live in fear and with feelings of being let down by the family courts (Birchall & Choudhry, 2018; see also Messing et al., 2021). Herman wrote in 2005 that the US justice system, both criminal or civil aspects, provides a context where the “wishes and needs of victims are often diametrically opposed to the requirements of legal proceedings” (Herman, 2005, p. 574), where women in particular are unlikely to be believed and are deemed to be fueled by revenge (not justice) when they report domestic violence or rape, and that “the victim's vision of justice is nowhere represented in the conventional justice system” (Herman, 2005, p. 574). In response to such concerns and the attempt to improve criminal and civil justice responses to GBV, there has been a raft of legislative changes in England and Wales with the tendency to further criminalize GBV. These have included the Domestic Violence Crime and Victims Act (2004), Equality Acts (2006, 2010), Crime and Security Act (2010), Forced Marriage Act (2007), Anti-Social Behaviour, Crime and Policing Act (2014), the Gender-Based Violence, Domestic Abuse and Sexual Violence (Wales) Bill (2014), and the Domestic Abuse Act (2021). The rights of victims have also gained specific attention through the establishment of the Victims’ Commissioner in 2013 and updates to the Victims’ Code in 2015 to comply with the European Union Victims’ Directive. The changing legislative landscape and shifts of all forms of GBV to be seen as crimes has provided a context where victims-survivors are more likely to seek criminal justice system intervention and may do so thinking these systems are “fair” (see Holder & Daly, 2018). However, reviews have continued to highlight serious problems in impunity in the response to victim-survivors of GBV, and identification of an ongoing “justice gap” in both domestic abuse and sexual violence cases (HMIC, 2014; HMICFRS, 2016; ONS, 2020; Stern, 2010). Moreover, attempts at improving post-separation family court approaches for victims through identification of domestic abuse have had little or no effect (Birchall & Choudhry, 2018).

These issues and context led providers of specialist support to victims-survivors of GBV in the UK to ask, “how do victims-survivors of gender based violence (GBV) currently experience and perceive justice,” thus creating the starting point for this research. Given that justice may be experienced via engagement with various criminal, civil, and/or family justice systems, and that victims-survivors of GBV may also hold notions of justice that go beyond the “formal” justice systems (Holder & Robinson, 2021), we decided to explore what “justice” means for victims-survivors of different forms of GBV and in the widest sense.

The findings presented in this article indicate that the dominant understandings of justice embedded within the legislative and policy approaches reproduce the “justice gap” by failing to take account of how survivors themselves understand and demand justice. Our findings are drawn from an in-depth analysis of qualitative data collected from interviews with 251 participants, all of whom had experience of some form of gender-based violence. This formed part of a larger research project centered on *Justice, Inequalities and Gender-Based Violence*.

## Background

Theoretical and empirical analyses of “justice,” whether in relation to the response of the police or the wider criminal or civil justice systems, or in relation to victim-survivors” perspectives, have been at the forefront of research about domestic and sexual violence since the 1970s (e.g., Holder, 2018; Radford, 1987). Alongside the provision of emergency refuge services, campaigners and researchers sought to improve the police and court responses to both protect women and children, and to increase the effectiveness of justice agencies. As indicated above, the past decades have seen considerable changes in the policies and practices in the UK (and elsewhere), which have also included provision of information and support for victims, the introduction of specialist domestic violence courts, and dedicated police units for domestic and sexual violence across a number of countries. However, in the past decade austerity measures in England and Wales have also led to a reduction in criminal justice staff and magistrates’ courts as well as specialist courts (Institute for Government, 2020), and undermined the ability of the criminal justice system to secure charges or convictions. Victim surveys have identified the high percentage of victims (particularly women) in domestic abuse cases who do not want to press charges; and the poor responses to women who report gender-based violence is apparent in recent attrition rate studies (see ONS, 2020; Walker et al., 2019). Although there has been an increase in victim-survivors of sexual violence who report to the police, many also feel revictimized and let down by the courts in particular (Hohl & Stanko, 2015). In this respect research from England and Wales echoes that from other countries, such as the US, where victim-survivors have reported feeling revictimized by criminal justice personnel and processes, and research has seen continued focus on “ideal” and stereotyped conceptions of victims by criminal justice personnel, especially in cases involving sexual violence (Carbone-Lopez et al., 2016; Murphy & Barkworth, 2014; Ricciardelli et al., 2021; Spencer , 2018).

Criminalization in particular has therefore been contested as an effective approach to tackling domestic violence, rape and other forms of gender-based violence. Feminists have highlighted the tension between a focus on often individualized criminal justice interventions and the potential detriment to women's empowerment, and understandings of gender inequality that may result (Herman, 2005; Mills, 2003, 2005; Russell & Light, 2006; Stark, 2007; Walklate, 2008). For instance, women's right to protection may have increased but the power and control she experiences from her partner may have merely transferred to the criminal justice system (Walklate, 1995). The contradictions in the usefulness of criminal sanctions have also led to more generic criticism about the appropriateness of criminal and legal remedies to gender-based violence more widely and raised questions about how and why individual victims-survivors access such sources of help, whether they achieve their intentions, let alone whether victims-survivors see them as “just” (Holder & Robinson, 2021). This is even more problematic for Black, Asian and Minoritized women where the relationship to the criminal justice system involves race (racism) as well as gender (sexism) (Thiara & Roy, 2020).

Of course neither formal nor informal justice systems are, or can, be independent of the culture and social mores within which they develop and through which they are administered (Walklate, 2008). As Walklate observes, talking in this instance about criminal justice, even when policy makers harness the progressive or symbolic function of law, for example promoting equalities, its translation through the criminal justice system is not assured. The prevailing social and cultural context is implicated at each point: from the victims-survivor's interpretation of what has happened; to their decision whether or not to disclose their experience of GBV; to the assessment of police and prosecutors on whether there is sufficient evidence to proceed; to the response in court of juries and/or magistrates/judges; to perpetrator's criminal actions themselves which can be enabled by wider sexual inequality and gendered norms. The gendering of criminal justice, for instance, can be seen in the differential treatment of women and men who report experiencing domestic violence and abuse. Research in England found that where men were deemed victims and women the perpetrators in domestic abuse cases, women were three times more likely to arrested than in instances where it was a female victim with male perpetrator (Hester, 2006). The gendering of justice in family courts has also been documented in numerous studies, where women may find it difficult to have their experiences of domestic abuse, and impacts on children, heard and accounted for (e.g., Lapierre et al., 2020; Radford & Hester, 2006; Stark, 2007).

Of direct relevance here are also other intersectional aspects. Research has found that individuals identifying as lesbian, gay, or bisexual (LGB) or transgender (T), are less likely to access the criminal justice system due to concerns about discrimination, although GB and T individuals may for instance experience higher rates of DVA than heterosexuals (Donovan & Hester 2014). Other aspects such as mental health impacts for the victim-survivors, have also been found to negatively influence the progression of domestic and sexual violence cases through the justice system, with such cases rarely proceeding beyond arrest or to conviction (Walker et al., 2019). Findings from other aspects of our wider study also demonstrate the different ways in which inequalities linked to “vulnerabilities” impact on the possibility of formal justice being achieved. Women from Black, Asian and Minoritized (in the UK) communities have reported experiencing the justice process differently from white women, with impacts of structural inequality, immigration status, and community and faith institutions playing into, and complicating the picture to different extents for different individuals (Aghtaie et al., 2020; Gangoli et al., 2020).

A systematic review of the literature was conducted by the research team as an early part of the wider research project, to explore wider forms and links between “justice,” “inequalities,” and “gender-based violence.” The review culminated in 38,119 hits which after sifting resulted in 1217 items which were included in the final review (Mulvihill et al., 2018). Mulvihill et al. (2018) and Mulvihill and Hester (2021) outline these different approaches to justice and the nuanced differences, and complex overlaps, between them. Of particular relevance here is the finding that only 20% to 25% of the items identified during the searches documented “the experiences and understandings of justice as articulated by victims/survivors of GBV themselves,” and thus researchers were often interpreting the perceptions and understandings of victims-survivors in relation to “justice” without attempting to elicit or directly facilitate their views (Mulvhill et al., 2018; Mulvihill & Hester, 2021). Our review also found that existing work on justice tends either towards a focus on process (procedural justice) or outcome (work on CJS outcomes for victim-survivors), micro-level (affective justice and empowerment) or macro-level change (though the latter is much-less talked about). Murphy and Barkworth (2014) point out that victims generally (and not necessarily experiencing GBV) tend to see procedural justice (the treatment they receive from justice authorities) as more important than the outcomes they achieve, with better outcome further enhancing the overall experience. Fairness in treatment by the police may thus be paramount, deemed to consist of neutrality, respect, trustworthiness, and voice (Murphy & Barkworth, 2014, p180). However, others point out that the outcome may be more important than process for some GBV victims-survivors, such as those experiencing domestic abuse (Hickmann & Simpson, 2003), and women in particular (Kulik et al., 1996). Our review found that the previous research, whether on process, outcome, affect or change, tends to center on a particular “terrain” or “social location” within or through which justice is sought or articulated (e.g., “the community,” the criminal justice system). While such work has its merit in detailing the specificities of a particular “focus,” “level,” and/or “terrain” of justice practices, we suggest it tends to be less attentive to the ways in which these overlap and intersect (Mulvihill & Hester, 2021).

Our review also found authors considering alternatives to criminal, civil, and family justice systems as a means of attaining “justice” for victims-survivors of GBV that may perhaps help us to think about what more victim-centered approaches might look like. Authors talked about ideas linked to wider notions of women's rights or human rights frameworks of justice, or social justice to combatting gender discrimination and oppression and securing rights and freedoms for women and men (e.g., First, 2006; Perilla, 2012), and community-based approaches, such as restorative justice (e.g., Gavrielides & Artinopoulou, 2013). Restorative justice is often mooted as an obvious alternative to the punitive approaches of criminal and civil legal systems, ideally providing victims with the possibility of voicing the harm they have faced, and the perpetrator taking responsibility for that harm, using a community-based meeting or setting involving third parties (see Braithwaite, 1989; Kim, 2021; Richardson & Wade, 2010). These approaches have previously been criticized as reproducing many similar problems associated with criminal or civil justice systems and may replicate existing power relations, thus further undermining and revictimizing victims. As Herman (2005, p. 579) pointed out, “The concerns of victims are insufficiently represented, and the interests of victims may be easily subordinated to an ideological agenda, in this instance an agenda of reconciliation rather than punishment.” The police in England and Wales have also been found more recently to be adopting what they see as restorative approaches in domestic abuse cases in a way that undermines outcomes and are likely to disempower victims-survivors (Westmarland et al., 2018). However, there are emerging approaches elsewhere that build on specific community experiences, often drawing on indigenous, and minoritized, and colonial experiences, that may overcome earlier concerns (Kim, 2021; Richardson & Wade, 2010).

This article is intended to help fill the knowledge gap about “what is justice,” based on the perceptions and experiences of victim-survivors, by describing and presenting the different ways in which the victims-survivors we interviewed talk about what both generic and specific concepts of justice mean to them, and how victims-survivors perceive what is justice in its widest sense.

## Methods

This article is based on data collected from interviews with 251 victims-survivors with a range of experiences of GBV (domestic, sexual, and/or “honor-based violence”) in England and Wales, during 2016–2018. The participants were recruited through over 80 agencies and organizations, and were all self-selecting as having experienced some form of abuse, and the sample tends to mirror the demographics of individuals accessing the range of agencies and organizations. Of the sample: 93% were female; 44% were under the age of 25; 32% identified as belonging to an ethnic minority and 5% identified as LGB. Forty-seven percent self-identified as having a mental health issue; 11% noted a physical disability and 5% identified as having a learning disability.

The interview schedule underwent a detailed developmental process which included consultation with a panel of victim-survivors and GBV professionals, and informed by the models of justice identified through the systematic review (Williamson et al., 2021). Interviews were carried out over the telephone, in-person, and using other on-line platforms as requested by participants. The average length of interview was an hour.

The research project had approval from the University of Bristol Faculty of Social Sciences and Law Research Ethics Committee. The team also has extensive experience of conducting research in this area, and has published widely on ethical issues which arise when conducting research with victims-survivors of abuse (Abrahams et al., 2015; Williamson et al., 2005).

### Analysis

Once the interviews were transcribed, they were then coded using an ecological model framework which identified experiences at the different levels of individual, community, organizational, and social, while also taking into account both process and outcomes for the victims-survivors. The Council of Europe in their holistic framework for tackling gender-based violence adopt an ecological approach based on the work of Hagemann-White et al. (2010) to identify areas and arenas for action and change. We decided to adopt this model because it could help us explore the very complex material and situate the participants’ experiences and perceptions at individual, micro, and macro levels, thus making the material easier to interpret and to share findings with professionals.

Using this overall process transcript summaries were produced including the coded data for that particular transcript. For the purposes of this article, we analyzed material contained in the summaries under the following two questions:

1. Putting your own experience to one side for a moment, what does the term justice/injustice mean to you?
2. And what would justice for you look like?

We categorized the material relating to these questions into key themes relating to the kinds of justice being referred to by participants. To represent victims-survivors perspectives on justice we used a theoretically informed but ultimately emergent/grounded secondary coding framework (Charmaz, 2005). To achieve consistency we coded the first 20 summaries and compared findings between team members. Through an iterative process of individually coding then comparing, then coding again, we identified and honed a set of themes or justice “types” which were found to relate to some of the broader themes identified within the literature from our systematic review, while retaining the emphasis given in the victim-survivors own accounts. We then coded the remaining summaries in accordance with these themes.

In terms of analysis it is important to recognize that this project interviewed participants at a single time point about any abuse they had experienced over their lifetime. As such, our findings bring together victim-survivors conceptions of justice from varying points in their “journeys” and combine multiple experiences, and shifting perceptions of “justice” for the individuals concerned.

## Findings

As indicated above, this article explores forms of justice sought by, or articulated by victims-survivors and some of the intersections between them. In attempting to typologize the diverse expressions of justice as articulated by victims-survivors it was necessary to bring these, often divergent, pockets of justice-thinking together in some way. We identified 642 mentions of forms of “justice” by the 251 participants. These clustered into the following themes: Accountability; Fairness (outcome)—procedural justice; Fairness in the process—Effective justice; Protection from future harm; Recognition—Being believed; Agency; Empowerment; Affective Justice; Reparation; and Social Transformation (see Table 1).

**Table 1.** Participants’ Views of Justice.

Themes	642 Responses from 251 Participants	%
Accountability	159	24.8
Fairness/equality (outcome)—procedural justice	100	15.6
Fairness (process)—effective justice	83	12.9
Protection from future harm—social justice	83	12.9
Recognition/being believed	60	9.3
Agency	37	5.8
Empowerment	36	5.6
Affective justice	29	4.5
Reparation	29	4.5
Social transformation—social justice	26	4.4

**Figure 1.**
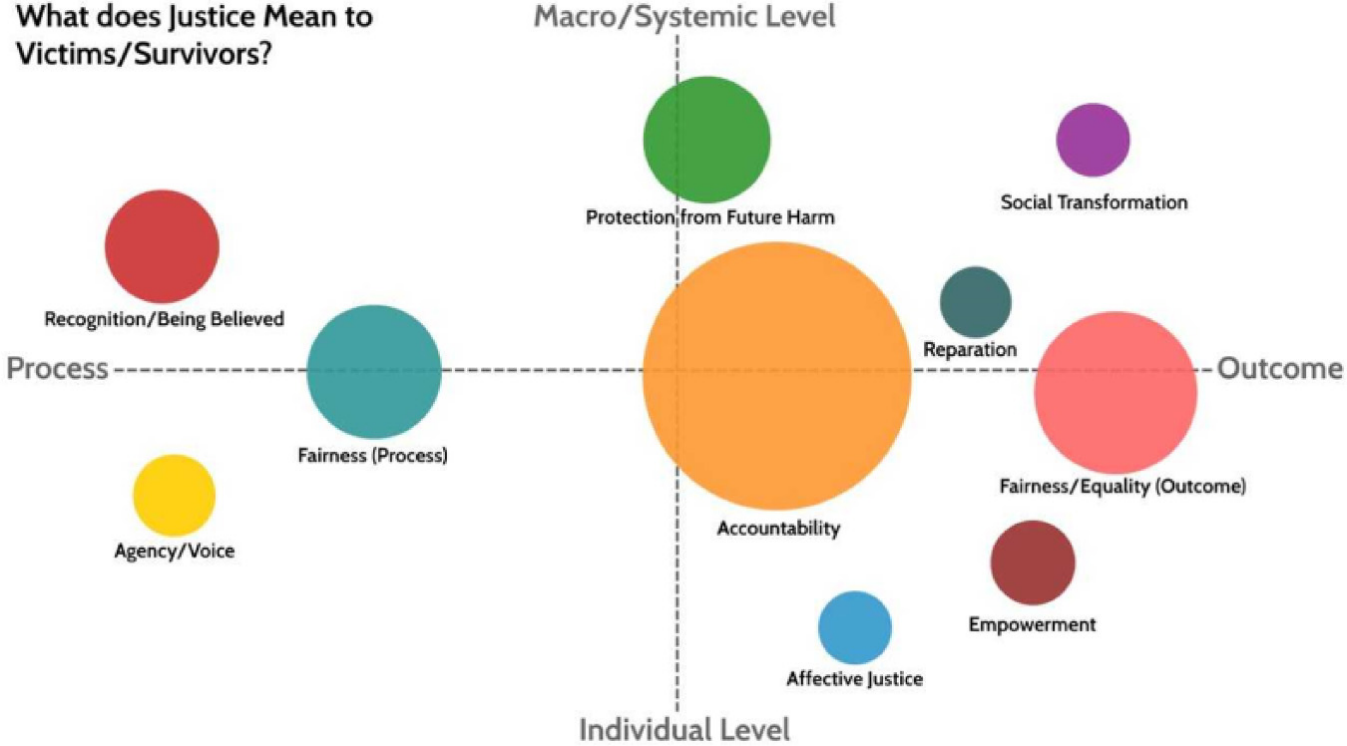
Overview of participants’ views of justice (*N* = 251).

For conceptual clarity, and specifically as a means to articulate the ways these forms of justice relate to one another, we mapped the themes identified onto a process-outcome diagram with a “macro-systemic vs micro” axis, and process-versus outcome axis (as illustrated in Figure 1). The rationale for the two axes were as follows:

1. Macro/Systemic vs. Micro axis
  This relates to the poles of Hagemann-White et al.'s (2010) ecological model and allows us to position justice types in terms of the degree to which they act, require action or are experienced at these levels.
2. Process vs. Outcome axis
  This refers to the degree to which the focus of this particular type of justice is centered on the justice process (or procedure, as in “procedural justice”) versus the justice outcome—that is what occurred in the end. The line between outcome and process is sometimes blurred, hence the utility of a continuous scale/axis.

Circle size, in figure 1, refers to the number of references to that particular justice “type” in the 251 coded summaries and circles thus represent the relative frequency of that justice type. As Figure 1 and Table 1 indicate, the largest category was “accountability,” with “fairness of outcome” second largest. The positioning of the circles was derived qualitatively through a combination of theoretical reflection and the content of the material itself. Thus, where theoretical reflection alone might lead us to position “accountability” as purely an outcome, analysis of the coded material revealed accountability to be composed of both process and outcome oriented elements. Crucially, many individual victims-survivors mentioned more than one form of justice and were thus located within multiple spheres. If a victim-survivor spoke about multiple justice types, multiple types would be coded (hence 642 responses from 251 participants). The categories are thus not mutually exclusive. This reflects victims-survivors complex relationships with justice at different points in their journeys and in terms of their experiences both of abuse, and attempts to get justice. This mirrors other work in this field (Cattaneo & Goodman, 2015; Holder & Daly, 2018) where the position of empowerment for example is complicated by the journey of victim-survivors through the formal justice processes. Moreover, the forms of justice talked about by participants were situated at individual, community, and societal (macro) levels.

The following sections discuss these findings starting from process and moving to outcomes, echoing the pattern in Figure 1 (i.e., beginning with the theme “agency”), and using quotes from participants to illustrate key areas they talked about. With regard to the largest category, “accountability,” we include more detail regarding sub-themes by using sub-headings.

### Agency

Having a sense of agency is the crucial first step for victims-survivors to have their views taken into consideration and whether they are able to meaningfully participate in the justice process. Victims-survivors’ agency is a central component of procedural justice, understood here as the degree to which a victim-survivor is able to have agency, a sense of self, in order for their experiences to be heard. For example, if a victim-survivor has been told repeatedly that the abuse did not happen, or has been undermined, they may not be able to embody their own knowledge and agency as a victim-survivor. Not being in a position to acknowledge the abuse happened denies even the possibility of justice, as recognized by those who talk about epistemic injustices (Fricker, 2007). As Herman (2005) points out, the requirements of legal proceedings “are often diametrically opposed” (p574) to what victims-survivors need or want.

Our participants talked about their agency and control over their lives, and decisions being taken away by both perpetrators and justice systems, although engaging with criminal justice also afforded agency and control to others. Asked about whether their idea of what justice is had changed as a result of their experiences, one of our victim-survivor participants responded,

Yeah definitely. […] Because I always thought it was just about like sentencing and … not just … it's more about like the emotional justice as well. It's like […] I just feel like my control's not just being taken by him, it's being taken by the people that are meant to protect me because they’ve made the decision for me. And in my eyes that's really unjust. (013)

Here then we see the importance of agency in the justice process specifically in terms of changing or redressing the dynamics of control that had been central to the harm experienced. More pointedly, in the words of another participant,

it's a way of taking back control I think, and kind of taking back your life to just say like “That was not okay.” And I think that's something that's been quite useful for me. (234)

These quotations illustrate the importance of victims-survivors having agency, and the connections they make between the controlling tactics of perpetrators and their potential lack of agency within the justice system (see also Walklate, 1995). In this sense agency in the external realm reinforced an ability to affect change in oneself and have some control over that change. Within the context of GBV where abuse is often linked to controlling and manipulative behaviors which undermine a sense of self within the victims-survivors, agency becomes paramount (Herman, 2005; Stark, 2007; Williamson, 2010).

### Recognition: Being Believed

Recognition and agency are closely linked. We separated them in order to signal the difference between acknowledging one's abuse, being heard, and having the ability to intervene in the justice process (as agency), and being believed or recognized as having been harmed, either by justice professionals, agencies, friends, family, or the perpetrator. Thus for the following victims-survivors, recognition from family and/or justice systems voicing their experiences and concerns was central to their sense of justice being done,

Whether it's the justice system or family members. … It's acknowledgement you’re not lying. And it's knowing that you were telling the truth and having your voice being believed and amplified. (140).

I think that belief has to come from more than just the professionals working with you. Because even though … you know even though my IDVA [Independent Domestic Violence Advocate] told me that she believed me, and even though my ISVA [Independent Sexual Violence Advocate] told me, it was like “Yeah, but you’re paid to tell me” you know “You wouldn’t be in the job that you’re doing … if you told me that you didn’t believe me you’d be fired’ you know so it kind of didn’t have that … it didn’t hold any weight for them to say to … I mean obviously it did, but you kind of need that … you need people … somebody who … a system that is not directly involved with your case that's telling you yeah we do believe you, we believe these things. (001)

Crucially, as we see in this second quote, recognition is differently weighted or valued by the victims-survivors in accordance with the degree to which it is expected, and/or the organization or individual offering recognition/belief is viewed by the victim-survivor to be impartial and/or powerful. The extension of this, of course, is recognition by others of harm done to the victim-survivor by the perpetrator—itself a key component in victims-survivors’ understandings of accountability-as-justice (discussed shortly). Recognition might also be linked to a victims’ rights approach where victims-survivors are enabled to give evidence by using special measures (e.g., providing evidence from behind a screen) because of the fear of intimidation by the perpetrator.

### Fairness in Process—Effective Justice

For many of the victims-survivors in this research justice related to fairness in the formal and informal processes of justice. This was the second largest category. Indeed combining the categories of fairness in process and fairness in outcome provided the largest number of responses overall regarding “what is justice,” thus also replicating the main concerns in our literature review with procedural justice and outcomes.

I think justice [is] about ensuring that you are keeping in tune with what the people want to make fair right decisions for your community and for those individuals within it to keep them safe and ensure that any wrongs are addressed fairly and investigated thoroughly (096).

This participant summarizes this idea of justice and fairness in a way that was common throughout the interviews. Central here is location of justice within or in relation to “community”—that fair decisions are judged not solely on their appropriateness for an individual but for all they impact on. This participant goes on however to identify the ways in which this fairness might not happen and why, based on identification of the perpetrator and judge as part of the same (male) group:

I think justice as I say should be fair, and representation to the community of doing what is right for individuals within that and ensuring that everything is fully investigated. But you can do all the investigation you like—when you’re up against a judge and actually they see that they’re wearing the same watch as the perpetrator and feel a bond there—that can be enough to tip them over into making a different decision. (096).

This participant is thus making a connection between the perpetrator and those enforcing justice alluded too through gender and class solidarity. This participant is questioning whether the male judge is aware of this potential bias and how that might impact implicitly on their decision-making. This issue of bias was also deemed important to participants in terms of race and ethnicity (Mulvihill et al., 2019).

A related reason why victims-survivors thought the process unfair related to vulnerability. Victims-survivors themselves would raise the question of how other victims-survivors, possibly positioned as more vulnerable due to issues of mental ill health (perhaps as a consequence of the abuse they experienced) or with lower levels of social or economic resources, could possibly negotiate their way through the criminal justice system. Police and other data examined in a different part of this project found that having a vulnerability^2^ was significantly associated with whether or not a case was likely to progress through the criminal justice system (Gangoli et al., 2020; Walker et al., 2019). They concluded, supporting the views of many victims-survivors we interviewed, that those with the most vulnerability are least likely to achieve justice.

### Affective Justice

For many of the victims-survivors we interviewed “justice” was something felt, experienced, embodied—or affective—rather than something which could be fully rationalized. We use the term “affect” here rather than emotion, seeing affect as the pre-cognitive embodied response to a stimulus while emotion is the naming and compartmentalization of that feeling—and is hence, post cognition (Massumi, 2002, p. 28). Thus justice/injustice elicits an embodied response that cannot be reduced to an existing category of justice that has been rationally defined. In the interviews, this “sense” of justice overlapped unevenly with other experiences and conceptualizations of what justice was or could be. Thus, agency, accountability or restoration might have an affective dimension—that *feeling* of justice being done that can accompany these justice types but is not necessarily linked to any one of them.

Okay so I think justice is anything which you feel right about. I think justice is something which you agree with, which makes you feel comfortable, which makes you think that you’ve been fairly treated, there is transparency, there is some proof to say it's been consistent, and there is no emotional, physical or any kind of threat attached to the outcome of any situation that you feel that you can be subjected to if you were to comply or non-comply with a particular thing. So I think justice is something that you feel comfortable with ethically, and um … without being subjected to any kind of threat. (040).

### Reparation

Reparation is doing something to repair the harm resulting from the abuse and may thus involve something offered to the victim-survivor by way of compensation, such as financial/economic compensation, (re)gaining access to housing, having therapy. Reparation thus also involves “distributive justice,” i.e., a socially just allocation of goods and resources, and “economic and financial justice” i.e., recognition of economic loss (housing, job, credit status, standing in community, confidence) inflicted by experience of GBV, and specific tactics of financial control. Our participants talked about these different aspects of justice, and especially the difficulties of obtaining these.

Victims-survivors who had experienced domestic abuse were especially concerned to have “financial justice” following the creation of debt and their money being taken by the perpetrators, and this was the key to their attempts to rebuild their lives. But they often found that banks and other agencies did not understand what had happened and thus undermined this route to justice. Victims-survivors were also told about compensation schemes, but such “distributive justice” carried risks of further injustice. For instance, one woman who had been raped saw her abuser convicted and was advised that she could claim compensation. However, incorrect information initially provided by the police to the compensation board resulted in her claim being rejected, thus causing unnecessary stress where she should have had financial justice: “I set out initially with the injustice of the abuse happening in the first place and then and I’ve ended up with another injustice” (17G).

### Protection From Future Harm—Social Justice

Whether the demand that abuse stop now, or that a victim-survivor, their family, friends or others yet-to-meet the perpetrator are protected from harm in future, protection from harm was a basic and necessary component of justice for many victim-survivors, and one that complicates an easy division between justice process and outcome.

For many participants, who had experienced domestic abuse, the ongoing nature of abuse post-separation was a source of injustice to them especially where they had engaged with the CJS with the expectation that this would put an end to the abuse.

[Do you feel like you got justice?] I suppose in a way yes, but in some ways no. I suppose I looked at it you know wrongly in the hope that this would all stop it, and it didn’t. So that's what's left me disappointed and upset. Whereas you know the system can only help you so far, [it's] not going to suddenly change someone and change their personality and how they are. So in some ways yes, in some ways no. (058).

For those victims-survivors who had children, there was considerable concern about how the child contact context was being used and manipulated by perpetrators in the family courts to continue their abuse, echoing numerous other studies (Birchall & Choudhry, 2018; Hester 2011; MoJ, 2020; Thiara & Harrison, 2016; Women's Aid, 2016).

the value of the rights of the father is put above everything else. And you can couch it in … you know the children's got a right to have … and the fact that they’ve even just changed the bloody … the Act to emphasize that the children should have a right to see both parents, it's just … it beggars belief for me, and I find that that's not justice when the people administering what justice is are ignoring all advice, then that's not justice. (049)

The ways in which child contact arrangements were being played out in domestic abuse cases felt to many participants as a form of injustice. Many felt that their ex-partners were using the contact as a means to punish them and inflict further abuse. Some suggested that the father had only become interested in the children once the separation had become a reality and they were fearful that this would be harmful for the children (see also Women's Aid, 2016). This often led to victims-survivors seeing justice as being about the protection of their children from that additional harm.

I think justice for me is that he wouldn’t be allowed to have anything more to do with the people that he hurt, such as me and our son … And it's not sort of being spiteful and trying to take him away, I think he shouldn’t be allowed to do what he did, and he shouldn’t be allowed to still have contact with those people (078).

Where victims-survivors perceived that injustice had taken place they were often concerned that this would allow the perpetrator to go on and abuse others. In this sense justice was perceived as a collective responsibility of the state to protect the public. This impact on others also extended to how victims-survivors thought about the wider social context in which abuse takes place. Some expressed concern for women generally about how vulnerable they are if an abusive partner comes along and decides to destroy what they have achieved.

and yet when you’re a young woman … cos sometimes l look at younger women and I think about myself […] but I look at younger women and they’re going around … and I just think God you don’t realize how vulnerable you are. Because they’re going around like confident, laughing, you know they buy themselves a car, they get a job … you know and I just feel like … it worries me because I think about my own child and I think well you know you can build your life and have a career, work hard, and yet some bloke can come along and just take that down (098).

This quotation links protection from future harm to wider gendered social structures which impact on the ways in which sexism and misogyny can operate. Vulnerability here is linked to how sexism can function in individual relationships to undermine the independence of women, with their own careers, property, and self-confidence, and that freedom can be taken very quickly (Women's Aid et al., 2021).

### Fairness in Outcome—Procedural Justice

For many victims-survivors, the lack of a positive formal outcome was perceived as an injustice. As was mentioned previously this related to both injustice for them, as well as fears about potential future harm to others. As Herman (2005) and others have also found, the disjunct between victims-survivors expectations of the formal justice outcome and the reality, was something which victims-survivors struggled to comprehend and come to terms with.

I mean I didn't know at the time, until the Court [case], but he was like a, a prolific pedophile. He'd raped kids before me and I mean obviously I wasn't raped I was just abused but he'd done a lot of stuff all over the country I think. But he'd always, whether he'd been caught or not I don't know but I think the times maybe he had been reported, he'd got away with it, you know, they'd put him on some sort of rehabilitation thing or, never been to jail. … You know, how can you not, how can you not be jailed for rapin’ a child? you know what I mean? (261).

This issue of expectations, sometimes in the literature referred to as victim satisfaction, has been reported elsewhere (Laxminarayan et al., 2013; Stern, 2010). What it highlights are the ways in which abstract concepts of what justice is, do not always fit with the procedural process of our criminal justice system. However, as the following quote indicates, where the victim-survivor had clear expectations of what was and was not possible in the criminal justice system, for instance, the experiences were more positive.

I'm really glad he got a prison sentence. The police hadn't lied to me; they'd said all along, ‘With regards to the rape, it's really difficult’. Because even if I'd have done something at the time, like called them at the time, when there would've been more evidence, they said because I'm married to him, or if you're in a relationship with somebody, it makes it a lot more difficult. Because people tend to think of people being raped as it's a stranger up a dark alley. They said only 6% of cases that go to court get convicted like that. (271).

This was one of the few cases where the perpetrator received a prison sentence in terms of outcome, but even without such outcome the victim-survivor suggests she appreciated the candid information she received from the police. This also links to the importance of advocacy support and information as elements in procedural justice and highlights positive outcomes for the victims-survivors we interviewed often came from informal sources outside the formal justice systems.

Participants also raised concerns regarding the way in which the formal processes do not address the long term and profound impacts that GBV has on victims-survivors, thus furthering the sense that outcomes are unjust.

### Social Transformation—Social Justice

Social transformation, linked individually to the protection of others from future harm in individual accounts, was evident across the participant interviews. Victims-survivors ultimately wanted to live in a world, in communities, families, societies where abuse did not exist. This led many of the participants (*N* = 72/251, 28.7%) to get involved in organizations, and/or politics either as volunteers, activists, or in terms of careers, as a way to fight for justice for other people, particularly where they didn’t perceive they had got justice themselves.

Justice for me would be that anything like that becomes as unpalatable, you know to the public as hitting somebody [outside family] or drink driving, or any of those things that used to be acceptable, and now are absolutely not. Cos there's not a world in which that should be acceptable behavior. But as long as there are people like that and people that turn a blind eye to it … or don’t want to get involved or don’t want to know, then it will always go on … if it could be made to be so totally unacceptable that you know people would be horrified … it would really help, and that would be justice to me. You know that people aren’t allowed to get away with it. (035).

As with the concept of fairness, participants considered justice and social transformation as linked. Thus the victim-survivor quoted above talks about justice as the shifting of public views from seeing certain (abusive) behaviors as acceptable to being considered “unpalatable.” Participants did not just consider justice in an individualistic way but in terms of how their experiences of abuse and justice were informed by the wider social discourse. Getting individual justice was not seen therefore as something separate from living in a just society.

Finally, social transformation as a course of action and activism was not limited to victim-survivors. The following participant takes the issues of accountability (which are discussed below) to their conclusion by talking about the ways in which perpetrators and men need to engage with the issues as a form of social injustice.

So I guess like a form of justice would be him … like if he was really going to take that on board to reach out to the women and people that he has impacted and like genuinely apologizing. And more than that, like actually being an advocate. I don’t know, listening, like being willing to listen and create a space for women … yeah of like being more than just apologetic, but like actually “Fuck, I did some really shit things, treated some people really badly, how can I stop?” Like it's so prolific in our society, so prolific. How am I going to contribute to preventing that from happening. If he gets to a place … I mean I really doubt this, that he's already there … I don’t know I just hope … if he could get to a place where he could truly recognize that and share that learning with other men, then that would feel like a form of justice. (232).

### Accountability

The largest cluster of responses fell within the category of accountability, which was mentioned by about a quarter of participants (159/251). This bears similarities to Holder and Daly's (2018) respondents who were all motivated to engage with the criminal justice system to hold the abuser—the perpetrator of domestic violence against women—accountable in some way. Within our category of accountability, we identified a range of sub-themes, including (in descending order of magnitude): recognition by the perpetrator and/or of authorities or community of harm done; punishment and rehabilitation; revenge or retaliation; and divine or spiritual accountability. We discuss each sub-theme separately.

#### Recognition by the perpetrator and/or of authorities or community of harm done

This sub-theme of recognition of harm done by perpetrators and/or authorities was the largest within our “accountability” category. It is what Herman (2005) termed “acknowledgement and vindication” and was also expressed by the largest sub-group of her 22 respondents.

Among our participants a strong theme throughout the interviews was the importance victims placed on external recognition that specific harm had been done to them. This was very often the first response to the question “what is justice?” and, for many, overrode ideas of punishment or retaliation:

…he doesn’t accept that there's anything wrong—and that isn’t justice to me. Justice would have been a realization on his part that what he did was utterly dreadful and the impact it had was utterly dreadful—that would be justice to me. You know that he’d get in touch with me and he’d sort of say “I’ve realized, I know now” *(035)*

It was important for recognition to come from the perpetrator themselves. This reflects the dynamics of GBV, in which the perpetrator often denies or minimizes their actions and the harm they have done, and where this reaction can be condoned by wider communities. Participants identified this pattern of minimization of behavior when asked to define “injustice”:

that person … does something wrong but then tries to put the blame onto the person they’ve actually done wrong by, or tries to manipulate them that makes it seems like they’re the bad one, they’re the one that's done the bad thing. Rather than taking responsibility and admitting what they’ve actually done. *(077).*

For many victims, as illustrated above, this recognition of harm needs to come from the perpetrator themselves and involve a genuine apology and expression of remorse. But in many cases this had not happened. While skillfully managed restorative justice approaches may offer spaces where victims-survivors can express the harm they have experienced and perpetrators own up to the harm they have done (Richardson & Wade, 2010; Kim, 2021), our participants appeared to have little experience of such approaches specifically. However, as the following quote illustrates, some participants were concerned that the perpetrator would merely “inch sideways” and avoid publicly taking responsibility for their harmful behavior.

Because I remember when kind of he was trying to make his way back here a couple of times, and it was very close to be successful at one stage, I remember one of my conditions—you can’t move back here until you tell your family what you’ve done. And he just inched that sideways, sideways, sideways “Of course I will tell them […]” and to the extent where then “No I cannot … you can’t even come into my house, in my mother's house, and mention anything about this ever again.” (011).

Where perpetrators did not acknowledge the harm they had done, the next best thing was for another party (the state, the police, their friends and family) to offer this recognition, and to hold the perpetrator (rather than the victim) responsible. For some participants this came from a formal criminal justice outcome, but for others it was the act of accountability through recognition that was important.

it's not necessarily the 10 years or 15 years, but rather the fact that your pain is legitimized and you’re heard. I think that's what a conviction gets for you. It doesn’t necessarily undo something, but it legitimizes your pain. (168)

Community was an important idea throughout the research interviews when participants were discussing what justice meant, particularly in relation to the acknowledgment of the harms caused by perpetrators. For those participants who actively sought to be part of groups or communities where accountability processes were explicit there was an awareness that such community responses were central to justice. As the following participant articulates, this can include self-help groups where the important element is being able to share experiences and concerns with others:

that's why self help groups work, because they speak to you … I mean they’re all sat in a circle chatting. Which is … you know if you’re living day to day round a fire like I do quite a lot of, […] when somebody's being a dick you go “You’re being a dick”—cos it's there, it's apparent, everyone sees it—you’re not just locked away in weird little rooms having a fucking shit time, you know, everyone's there, everyone's part of each other … everything is obvious, everyone's got an input and a help and a procedure for like “Oh yeah, oh [Perpetrator] is kicking off again, let's go and you know … let's give him a spliff and chill out” you know […] But it doesn’t work if you’re shut away in little rooms all the time—that's when it gets weird—we haven’t evolved in little rooms, we haven’t. That's why my kitchen table is round … it needs to be round, cos everyone is part of the same shit. And equally … needs equally as much help. (232)

Family and community were also important in terms of future safety. By requiring the perpetrator to admit to family members what they had done, some victims-survivors were wanting the responsibility of knowing what he had done to be shared, sometimes linked to future safety, and for these family members to understand the harms and injustice they had been living within.

#### Accountability: Punishment and rehabilitation

Participants referred to both punishment and rehabilitation, often in conjunction with one another. Criminal sanctions which resulted in either were welcomed because they represented recognition of the harm by perpetrators (as above).

And these men … I personally do not believe that anything can be done until they’re given harsher sentences for domestic violence, nothing is going to change. If you get a suspended sentence for GBH [Grievous bodily harm], then what's that telling you? That's not saying stop, it shouldn’t happen is it? (008).

As with most of the participants who talked about formal punishment through the criminal justice system, there was concern that where perpetrators did not receive a criminal sanction this had further negative impacts on both them and the way society and others view this type of abuse.

Um, I guess it's sort of two fold really, it's whether it's sort of like a legal form of justice or a more kind of social form. It could be either sort of going to court and getting some sort of you know punishment for something, or conviction for something, or it can be sort of from the sort of peer group to the social setting around you, like family, friends and acquaintance—I think there's that sort of phrase isn’t there “having your just deserts” kind of thing—I think that comes under justice as well. (037).

For many victims-survivors, punishment was related more to the social community response of others. This links with the idea of justice being recognition, as discussed above. Given that formal conviction rates for GBV are notoriously low (Walker et al., 2019) it is not surprising that the majority of participants who equated justice with punishment felt let down by the formal system.

The frequency of participants talking about punishment and rehabilitation was not as unequal as one might have expected. For some victims-survivors, punishment was relevant because there was no accountability. For others rehabilitation was a form of social transformation located in the aim to prevent future harm both for themselves and others.

#### Revenge/retaliation

Herman (2005) suggests that women are perceived to engage with justice systems primarily to get revenge, but few of her respondents had this motivation. Our work echoes this. The idea of taking revenge on the perpetrator or retaliation was rare in the responses from our participants. What comes through in the findings is the way in which many participants were at pains to make clear that their attempts at seeking justice are not a form of retribution, retaliation, pay-back, revenge etc. It suggests that many participants think that their attempts at seeking any type of justice are seen by others as negative and vindictive. This suggests that even when discussing justice in the context of their personal experiences, the right to even seek justice is not a given for these participants.

Instead, participants were concerned about possible retaliation by others, and in particular that male family members might seek revenge on their behalf. This resulted in (female) victims-survivors not confiding in those individuals for fear of the consequences. They felt that if something were to happen then they would be to blame. Where revenge was raised it was generally discussed and then discounted. For example, one participant (069) discussed how her grandfather had offered £10,000 to “get rid” of the perpetrator which she didn’t want because it was “illegal.”

Some participants linked ideas of retaliation to divine retribution or karma (which will be discussed in the next section):

Last year I got word that he was in a really bad car crash and his lungs collapsed and he was in intensive care … I felt really good about it […] I thought you know what, karma works in so many ways. And all the pain he ever gave me came to him all in one go. (128)

This type of revenge, where something happened to the perpetrator but where the victims-survivors did not need to act was seen as a positive type of justice.

#### Divine or spiritual accountability

Alongside accountability and recognition from perpetrators, authorities, and the wider community, a number of participants also referred to divine or spiritual forms of accountability. Herman (2005) talks about “forgiveness” by the victim as a key aspect of religion and that it thus undermines accountability as justice. For our participants however there was a sense that “the divine” might act directly on the perpetrator, as “karma” or eventual “heavenly” retribution.

And then there's also I guess the kind of karma or what goes around comes around kind of justice, like natural justice. Like if you do wrong to somebody at some point it will be done to you, but that's not human, that's kind of a more of perhaps a spiritual or maybe a religious type of justice. Sometimes you see things happen and that … you think it's just so unfair, it's so unjust, somebody's not been sent to prison for long enough, or they’ve never been caught for what they’ve done. But somewhere down the line they’ll get justice—it will happen at some point. (038)

This quotation is typical of those who believed that where justice had not been served, karma or divine retribution was a possibility. For some this enabled victim-survivors to move on with their lives by accepting that justice might not have been served but that it would eventually.

I don’t know I think justice would be if my husband realized what he has done and he realizes what he has lost I think that would be my justice … and to tell you the truth I am not concerned about justice, I am more concerned about the justice we get after we die … so with our God and everything so I am more concerned about that really. (145).

For some participants however, their difficulty in accepting divine justice was perceived as a failure on their part. They felt that their inability to trust in a future justice was evidence of a lack of their faith. Further analysis of our findings regarding spiritual abuse and the response of faith communities is available elsewhere (Aghtaie et al., 2020) but it is important here to recognize that this type of justice elicited both positive and negative impacts on victims-survivors.

#### Empowerment

Empowerment in the context of GBV and justice is a complex concept and one that as Cattaneo and Goodman (2015) recognize is often used as a general catch all to refer to victim focused interventions, victim informed processes, as well as positive victim outcomes. The participants in our research used the concept of empowerment either directly or indirectly in all of these different ways. Empowerment was linked to the notion of agency already discussed. It was also used in terms of how victim-survivors felt as a result of the processes of trying to get justice through accountability and in relation to “others.”

So I don’t know in some ways I feel like justice is getting out of my head all of these people who have mistreated me, who are all somehow living inside of me still, you know. It's like it's getting some kind of boundary and some kind of “no” against them … and then with that a sense of worth, it's dignity. (120)

Empowerment was also evident when victim-survivors talked about the longer term processes of dealing with the impacts of abuse. Echoing Abrahams’ (2007) work on the longitudinal impacts for abuse victims, participants talked about empowerment being represented in the little things that they start to have control over once the abusive relationship had ended, or they had undergone some form of healing, for example: buying books, going to a gig, spending time with friends, or removing a huge TV.

As discussed above in terms of social transformation, we also found evidence in the research of how for many participants empowerment came through helping others. We know from previous research (Dobash & Dobash, 1992; Stark, 2007) that the GBV sector includes many activists who have experienced abuse (Gilbert, 2019). In fact, the early development of services for women experiencing GBV was about linking the personal and the political, empowerment and social change.

Empowerment was also important in terms of addressing the position of the victims-survivors outside of considerations of whether they got justice. Whether the perpetrator was held to account or not, punished or not, victims-survivors still have to move on and rebuild their lives following experiences of abuse. For many it is this process of escaping and surviving which was important and led to their empowerment.

## Conclusion

This article has presented the perspectives of victims-survivors of GBV on what justice means to them, that the experiences and perceptions of victims-survivors are complex, they may have more than one perception of justice, that these can relate to different points in the survivor's journey following abuse, and considered in relation to individual, community and societal responses. This has echoes with Holder and Daly's work (2018) where they argue that we need a take a longitudinal approach to justice with domestic violence victims.

The findings echo previous work on procedural justice, in particular the emphasis of our participants on fairness of process leading to outcomes that are fair and restrain the perpetrators’ ability to continue their abuse (Hickman & Simpson, 2003). Our work goes further, however, by looking beyond merely criminal justice, and indicates that fair outcomes are features that victims-survivors similarly deem as “justice” in relation to civil and family courts.

The picture that emerges from our findings is the need for a victim-centered justice where perpetrators are held accountable, with fair outcomes for victims-survivors leading to protection from future harm of themselves and others. Justice is deemed where survivors are heard and believed at all levels, where the perpetrator, families and communities as well as formal justice systems recognize and acknowledge the abuse so that there is accountability for the harm done. This recognition was important for victims-survivors in the process of feeling fairness, and ultimately being empowered, and in control of their own lives. Our findings show that when victims-survivors of GBV are asked about their perceptions of justice these range much further than merely formal systems such as engagement with criminal, civil or family justice professionals and processes, and they see families and communities as having important roles alongside, and especially where formal systems fail the victims-survivors. Given these findings we might have expected experiences of “restorative justice” approaches as alternatives to formal justice systems, but these were not obvious. Other work in England and Wales on advocacy with rape victims has indicated that practitioners have a “mender” role, to enable restoration of relationships between victims and their families (Hester & Lilley, 2017), but this does not involve the perpetrator.

The findings highlight the profound lack of justice that participants experienced, leaving them subject to or concerned about revictimization and blame by both formal systems and communities. Despite these very negative aspects, the victims-survivors in our large interview sample also showed how they were finding ways to create some positive outcomes and forms of “justice” for themselves, including engagement with social justice (First, 2006) in the sense of working to support others and other means of empowerment through activism. The justice perspectives and justice needs of the participants also resonate with the cultural context model, suggested by Almeida and Lockard (2005) as a model of accountability and empowerment to respond to domestic abuse, and which is rooted in principles of universal human rights and practices that foster a critical consciousness. As our research shows, individuals may be deemed to be acquainted, even tacitly, with the “rules” of different gender regimes (e.g., Walby, 2020; and see Walklate, 1995). As such, victims-survivors of gender-based violence understand that they may need to bridge the gap between the formal promise of law and the subjective reality of the criminal and other justice systems if they are to achieve justice. Participants were equally likely to refer to punishment and/or rehabilitation in a formal sense, but for many the accountability they wanted within families and communities were perceived as punishment enough, partly because this outcome was considered highly unlikely.

This article also highlights throughout how victims-survivors preface their views about justice with justifications as to why they are not seeking revenge, pay-back, retribution, etc. This suggests that we are not dealing with a level playing field, and that justice is not a universal given for victims-survivors of GBV when compared to other types of crime. These victims-survivors feel the need to make clear that their motives for seeking justice are not vindictive, even though they are victims of potentially illegal behaviors. It is difficult to imagine the victims of other types of crime feeling the need to justify their search for justice. This is indicative of the wider views of victims-survivors of GBV and the ways in which they are represented. These representations, which lead victims-survivors to feel the need to justify their rights to seek justice, are reinforced by the lack of accountability from perpetrators which victims-survivors seek. What victims-survivors are seeking is what Herman (1992, 2005) refers to as: action, engagement, and remembering. Perpetrators throughout the accounts presented here evaded justice in all its forms and we, as individuals, communities, professionals, and the justice system itself enable that to happen.

It is very tempting to take the side of the perpetrator. All the perpetrator asks is that the bystander do nothing. He appeals to the universal desire to see, hear, and speak no evil. The victim, on the contrary, asks the bystander to share the burden of pain. The victim demands action, engagement, and remembering. (Herman, 1992, pp. 7–8).

Thus, withdrawing from the criminal justice or civil justice system process, for example, could indicate positive, self-protective choices by victims-survivors who recognize the type of “justice” on offer is not what, or how, they want, and it may simultaneously be an indictment of the prevailing formal systems and raises the question of what alternatives are available (Hester, 2006). We have shown that the “justice gap” that victims-survivors perceive is much wider than merely formal criminal or civil justice systems, and that the gap will continue to exist as long as we fail to take account of and place at the center how survivors themselves understand and demand justice.

## Notes

1. SHOULD BE 2020

2. Vulnerability here might be an individual vulnerability in terms of mental health issues for example or structural inequalities related to ethnicity, immigration status, class, or sexuality.
